# Cryptic diversity in *Hipposideros commersoni* sensu stricto (Chiroptera: Hipposideridae) in the western portion of Madagascar

**DOI:** 10.1186/s12862-015-0510-2

**Published:** 2015-10-30

**Authors:** Andrinajoro R. Rakotoarivelo, Sandi Willows-Munro, M. Corrie Schoeman, Jennifer M. Lamb, Steven M. Goodman

**Affiliations:** School of Life Sciences, Pietermaritzburg Campus, Rabie Saunders Building, University of Kwa-Zulu Natal, Pietermaritzburg, 3209 South Africa; Association Vahatra, BP 3972 Antananarivo, 101, Madagascar; School of Life Sciences, Biological Sciences Building, South Ring Road, Westville Campus, University of Kwa-Zulu Natal, Westville, 3630 South Africa; Field Museum of Natural History, 1400 South Lake Shore Drive, Chicago, IL 60605 USA; Department of Genetics, University of KwaZulu-Natal, Pietermaritzburg Campus, Rabie Saunders Building, Rm 36, Pietermaritzburg, 3209 South Africa

**Keywords:** Dry forest, Phylogeny, Paraphyly, Evolutionary history, Systematics, Morphology

## Abstract

**Background:**

The Commerson’s leaf-nosed bat, *Hipposideros commersoni* sensu stricto, is endemic to Madagascar and is relatively common in the western portion of the island, where it is found in areas, including forested zones, from sea level to 1325 m. A previous study on morphological patterns of geographic variation within the species highlighted the presence of two distinct morphotypes; larger individuals in the north portion of the island and smaller individuals in the south. The main aim of this study was to use a combination of craniodental morphology and molecular data (mitochondrial and nuclear) to test previous hypotheses based on morphology and clarify the evolutionary history of the species group.

**Methods:**

We sequenced mitochondrial and nuclear genes from *Hipposideros commersoni* obtained from the western portion of Madagascar, and compared them with other African species as outgroups. We analyzed the sequence data using Maximum Likelihood and Bayesian phylogenetic inference. Divergence dates were estimated using Bayesian molecular clock approach. Variation in craniodental variables was also assessed from sequenced individuals.

**Results:**

The molecular analyses suggest that *H. commersoni* is not monophyletic, with strong support for the presence of several independently evolving lineages. Two individuals amongst those sequenced from Isalo (south central) and Itampolo (southwest) form a separate clade (Clade A), distinct from other *H. commersoni*, and sister to continental African *H. vittatus* and *H. gigas.* Within the *H. commersoni* clade, the molecular data support two geographically distributed clades; one from the south (Clade B) and the other from the north (Clade C), which diverged approximately 3.38 million years ago. Morphometric data were consistent with the molecular analyses, suggesting a north–south break within *H. commersoni*. However, at some localities, animals from both clades occurred in sympatry and these individuals could not be differentiated based on external and craniodental measurements.

**Conclusions:**

Using a combination of molecular and morphological characters, this study presents evidence of cryptic diversity in *H. commersoni* on Madagascar. Further fine-scale phylogeographic studies are needed to fully resolve the systematics of *H. commersoni*. This study highlights the utility of the combined approach in employing both morphological and molecular data to provide insights into the evolutionary history of Malagasy population currently assigned to *H. commersoni*.

**Electronic supplementary material:**

The online version of this article (doi:10.1186/s12862-015-0510-2) contains supplementary material, which is available to authorized users.

## Background

Members of the Family Hipposideridae, known as Old World leaf-nosed bats, are one of the most widespread and abundant groups of insectivorous bats and inhabit tropical and subtropical regions of Africa, the Middle East, Asia and Australia [1]. To a large extent, species within this genus have been defined based on their external and craniodental morphology. In a recent summary, 70 species of *Hipposideros* were recognized [1], subsequently numerous other taxa have been described (e.g. [2–5]) and the taxonomy of the group, predominantly at the species level, is far from resolved. As a tool to understand the evolutionary history of members of this genus, closely related species are often placed in morphological species groups (e.g., [1, 6]). As currently delineated, the *H. commersoni* group includes the Afro-Malagasy taxa *H. commersoni* (É. Geoffroy, 1813), described from Madagascar, and *H. thomensis* (Bocage, 1891), *H. gigas* (Wagner, 1845) and *H. vittatus* (Peters, 1852), from continental Africa and offshore islands. The last-named three forms were previously considered subspecies of *H. commersoni* sensu lato, but were recently raised to species rank [1] based on reputed morphology and echolocation call differences [7–9]). As members of the *H. commersoni* group s.l. have to date not been the subject of a detailed phylogenetic study, it is unclear if these taxonomic changes reflect the evolutionary relationships within this portion of the genus or are examples of morphological convergence [10].

*Hipposideros commersoni* sensu stricto is a widespread endemic to Madagascar and can be found from sea level to 1325 m, generally in forested zones [11]. Its diet (mostly Coleoptera) and activity in western Madagascar may change seasonally and may be related to possible intra-island movements [12–15]. On the basis of current information, Malagasy populations of *H. commersoni* demonstrate considerable geographic variation in morphological measurements and certain patterns cannot be explained by simple clines [16].

In this study, we examine genetic and morphological variation in *H. commersoni* using samples obtained from different areas of Madagascar within and outside dry forest formations, specifically the western half of the island, to explore aspects of their phylogenetic history and to help resolve the systematic relationships of the different morphotypes recovered by Ranivo and Goodman [16].

## Methods

### Morphological and molecular sampling

**Fig. 1:**
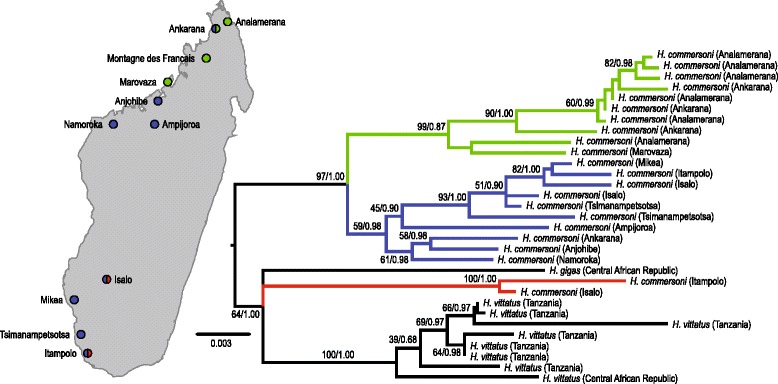
Geographical distribution of *Hipposideros commersoni* specimens analysed in the present study (*left*). Localities are colour-coded according to the main phylogenetic lineages (*red* = Clade A, *blue* = Clade B, *green* = Clade C). Maximum likelihood tree (*right*) inferred from the combined analysis of molecular data (two mtDNA [CR and *Cyt b*], two nuclear introns [bSTAT and OSTA5]). Posterior probability values and maximum likelihood bootstrap support (in that order) are shown at the nodes

In this paper, reference to *Hipposideros commersoni* is restricted to Malagasy populations and, hence, sensu stricto. A total of 22 *H. commersoni* (20 females and two males) were included in the molecular portion of this study (Table 1). During the past two decades intensive bats survey were carried out by different researchers across Madagascar, but with a distinct bias towards the west, where there are often extensive cave systems used as day roost sites for *Hipposideros*. The collection of *H. commersoni* specimens and associated tissues were greatly biased in this context. This species is present in eastern part of Madagascar but only a few specimens were available. Morphological analyses were only conducted on the 20 females. These samples come from collections made over the past 15 years from 11 localities across the western portion of Madagascar (Fig. 1). All voucher specimens are cataloged in the Field Museum of Natural History (FMNH) or Université d’Antananarivo, Département de Biologie Animale (UADBA). Samples used in the molecular study included the aforementioned material, as well as additional tissue samples of *H. vittatus* (*n* = 7) and *H. gigas* (*n* = 1), two morphologically similar species [17], from the FMNH and the American Museum of Natural History (AMNH) collections (Table 1, Fig. 1).

**Table 1:** Details of specimens included in the molecular analysis (*n* = 22, 20 females and 2 males). The *Hipposideros commersoni* specimens are all from Madagascar; more precise details on collection localities are presented in Appendix 1

Species	Museum number	GenBank numbers				Collection locality
		CR	*Cyt b*	bSTAT	OSTA5	
*Hipposideros commersoni*	FMNH 169707	KT371749	KT5838015	KT583770	KT437663	Andrafiabe, Ankarana
*Hipposideros commersoni*	FMNH 175777	KT371750	KT5838022	KT583771	KT437664	Andranomavo, Namoroka
*Hipposideros commersoni*	FMNH 175966	KT371751	KT5838023	KT583772	KT437665	Menamaty, Isalo
*Hipposideros commersoni*	FMNH175970	KT371752	KT5838011	KT583773	KT437666	Berenty-Betsileo, Isalo
*Hipposideros commersoni*	FMNH 176155	KT371753	KT5838024	KT583774	KT437667	Ankiloaka, Mikea Forest
*Hipposideros commersoni*	FMNH 177302	KT371754	KT5838025	KT583775	KT437668	Ampijoroa
*Hipposideros commersoni*	FMNH 178806	KT371755	KT5838016	KT583776	KT437669	Bazaribe Cave, Analamerana
*Hipposideros commersoni*	FMNH 178808	KT371756	KT5838017	KT583777	KT437670	Bazaribe Cave, Analamerana
*Hipposideros commersoni*	FMNH 178809	KT371757	KT5838018	KT583778	KT437671	Bazaribe Cave, Analamerana
*Hipposideros commersoni*	FMNH 178810	KT371758	KT5838019	KT583779	KT437672	Bazaribe Cave, Analamerana
*Hipposideros commersoni*	FMNH 178811	KT371759	KT5838020	KT583780	KT437673	Bazaribe Cave, Analamerana
*Hipposideros commersoni*	FMNH 178815	KT371760	KT5838021	KT583781	KT437674	Bazaribe Cave, Analamerana
*Hipposideros commersoni*	FMNH 178812	KT371761	KT5838026	KT583782	KT437675	Bazaribe Cave, Analamerana
*Hipposideros commersoni*	FMNH 183934	KT371762	KT5838027	KT583783	KT437676	Mitoho Cave, Tsimanampetsotsa
*Hipposideros commersoni*	FMNH 184170	KT371763	KT5838028	KT583784	KT437677	Androimpano Cave, Itampolo
*Hipposideros commersoni*	FMNH 184173	KT371764	KT5838010	KT583785	KT437678	Androimpano Cave, Itampolo,
*Hipposideros commersoni*	FMNH 184030	KT371765	KT5838012	KT583786	KT437679	4.2 km SE Marovaza, in cave
*Hipposideros commersoni*	FMNH 183980	KT371766	KT5838013	KT583787	KT437680	Ampitiliantsambo Forest, Montagne de Français
*Hipposideros commersoni*	FMNH 217940	KT371767	KT5838031	KT583788	KT437681	Ranohira, Isalo
*Hipposideros commersoni*	UADBA 32987	KT371768	KT5838014	KT583789	KT437682	Andrafiabe, Ankarana
*Hipposideros commersoni*	FMNH 221308	KT371769	KT5838029	KT583790	KT437683	Andrafiabe, Ankarana
*Hipposideros commersoni*	UADBA32916	KT371770	KT5838030	KT583791	KT437684	Anjohibe Cave, Antanamarina,
*Hipposideros vittatus*	FMNH 192800	KT371772	KT583803	KT583792	KT437685	Kasinji Region, Pemba Island, Tanzania
*Hipposideros vittatus*	FMNH 192857	KT371773	KT583804	KT583793	KT437686	Kasinji Region, Pemba Island, Tanzania
*Hipposideros vittatus*	FMNH 192858	KT371774	KT583805	KT583794	KT437687	Kasinji Region, Pemba Island, Tanzania
*Hipposideros vittatus*	FMNH 192859	KT371775	KT583806	KT583795	KT437688	Kasinji Region, Pemba Island, Tanzania
*Hipposideros vittatus*	FMNH 192860	KT371776	KT583807	KT583796	KT437689	Kasinji Region, Pemba Island, Tanzania
*Hipposideros vittatus*	FMNH 192865	KT371777	KT583808	KT583797	-	Kasinji Region, Pemba Island, Tanzania
*Hipposideros vittatus*	FMNH 192866	KT371778	KT583809	KT583798	KT437690	Kasinji Region, Pemba Island, Tanzania
*Hipposideros gigas*	AMNH 269871	KT371748	KT583801	KT583799	KT437691	Dzanga Sangha Forest Reserve, Central African Republic
*Hipposideros vittatus*	AMNH 269879	KT371771	KT583802	KT583800	KT437692	Dzanga Sangha Forest Reserve, Central African Republic

Collection numbers are those assigned to each specimen by museums FMNH (Field Museum of Natural History), AMNH (American Museum of Natural History) and UADBA (Université d’Antananarivo, Département de Biologie Animale; − = missing data

### DNA sequencing

Genomic DNA was isolated using the NucleoSpin® Tissue kit (Macherey-Nagel, Germany), following the manufacturers protocol for tissue samples. Two mitochondrial (mtDNA) and two nuclear intron (ncDNA) markers were amplified.

The cytochrome *b* gene (*Cyt b*) was amplified using two sets of nested primers. The primers L14724AG (5′–ATG ATA TGA AAA ACC ATC GTT G–3′; [4]) and H15915 (5′–TCT CCA TTT CTG GTT TAC AAG AC–3′; [18]) were used to amplify a 1200 bp segment of *Cyt b*. In specimens in which L14724AG and H15915 did not amplify, the primers JorF (5′-GAC CTT CCA ACT CCC TCA AGC AT-3′; designed for study) and H15553 (5′–TAG GCA AAT AGG AAA TAT CAT TCT GGT–3′; [18]) were used to amplify a smaller 700 bp segment. The hypervariable portion of the control region (CR) of the mitochondrial genome was amplified in all specimens as a single fragment using primers P (5′–TCC TAC CAT CAG CAC CCA AAG C–3′) and E (5′–CCT GAA GTA GGA ACC AGA TG–3′; [19]). The 16^th^ intron of the signal transducer and activator of transcription 5A (STAT) was amplified using previously published primers (bSTATa 5′–GAA GAA ACA TCA CAA GCC CC–3′, bSTATb 5′–AGA CCT CAT CCT TGG GCC–3′; [20, 21]). The 5^th^ intron of the organic solute transporter subunit alpha gene (OSTA5) was amplified using the primers OSTA5F (5′–TGM WGG YCA TGG TGG AAG GCT TTG–3)′ and OSTA5R (5′–AGA TGC CRT CRG GGA YGA GRA ACA–3′; [22]). The STAT marker was used based on the work of Eick et al. [20], who found high levels of intraspecific divergence for this marker in 58 bat species. Igea et al. [22] identified the intron OSTA5 to be an adequate marker for analyses of species delimitation, gene flow and genetic differentiation within two bat species. Cycle sequencing was performed using BigDye Chemistry (Version 3.1, Applied Biosystems, USA), and products analyzed on a 3100 ABI automated sequencer (Applied Biosystems). All heterozygous sites in the ncDNA were coded using the IUB code. All sequences were first aligned using ClustalW [23] as implemented in BioEdit [24], and thereafter manually to optimize homology. All new sequences were deposited in GenBank (Table 1).

### Sequence analyses

The four markers (CR, *Cyt b*, STAT and OSTA5) were analyzed separately and then combined into a single data set. Gaps were treated as missing data. In addition, the markers were concatenated and analyzed according to origin of marker (mtDNA or nucDNA). The number of variable sites, number of parsimony informative sites and nucleotide frequencies were estimated for each data matrix in MEGA 6 [25].

Phylogenetic reconstruction was performed using both maximum likelihood (ML) and Bayesian (Bayes) approaches using the programs Garli 2.0 [26] and MrBayes 3.2 [27], respectively. The most appropriate substitution model for each gene (CR - HKY + I + G, *Cyt b*- HKY + I, STAT - TIM1 + I, OSTA5 - TIM1ef + I) was selected using the Akaike information criterion (AIC) as implemented in jModelTest [28, 29]. For the concatenated data sets, partitioned analyses were conducted, with data partitioned by gene, with the parameters of nucleotide substitution models unlinked across partitions. Each ML analysis was initiated from a random starting tree, with nodal support assessed using 1000 bootstrap replicates. Two independent Bayes runs of 5 million generations each were performed; each run consisted of four Monte Carlo Markov chains (MCMC), with topologies sampled every 250 generations. The program Tracer 1.6 [30] was used to determine that the effective sample size (ESS) had reached > 200 for all parameters. A 50 % majority rule consensus tree was constructed using the CONSENSE program in the PHYLIP package [31]. In each simulation the first 20 % of generations were discarded as burn-in, after a pilot run to determine that this was sufficient to achieve stationarity.

### Molecular dating

No Rhinolophidae or Hipposideridae fossils are known from before the middle Eocene, but fossils referable to both families are reported from the middle to late Eocene of Europe [32, 33], including *H. schlosseri* from the late Eocene of France [34]. As fossil calibration points are not available for *H. commersoni* s.l., we expanded the taxonomic sampling used in the molecular clock analysis to allow the use of fossil calibration points. *Cyt b* sequences were downloaded from GenBank for six species of *Hipposideros*: *H. armiger* (DQ865345), *H. pratti* (EF544427), *H*. aff. *ruber* (EU934485), *H*. aff. *caffer* (EU934461), *H. gigas* (EU934469) and *H. cyclops* (EU934466), as well as eight species and 12 individuals of the family Rhinolophidae considered as sister to the Hipposideridae [35, 36]: *R. mossambicus* (JQ929291, JQ929299), *R. eloquens* (JQ929284, JQ929285), *R. hildebrandtii* (JQ929297, JQ929298), *R. darlingi* (EU436675), *R. fumigatus* (FJ457614), *R. landeri* (EU436668, FJ457612), *R. ruwenzorii* (EU436679) and *R. maclaudi* (FJ185203). As calibration point, we used a minimum of 37 Mya and maximum of 55 Mya for the split between the Rhinolophidae and the Hipposideridae [20, 37, 38].

Divergence dates between clades were estimated from the expanded *Cyt b* data set using an uncorrelated relaxed lognormal Bayesian molecular clock approach [39], as implemented in BEAST 2.1.3.0 [40]. The HKY + I substitution model was used, with the Yule speciation model as tree prior. As an alternative to fossil calibrated estimate of divergence times, an additional molecular clock analysis was conducted using a fixed mean substitution rate of 1.30 × 10^−8^ subs/site/year [5, 41]. This analysis was performed using the strict molecular clock model in BEAST. All other parameters were the same as in previous analysis. The MCMC chains were run for 30 million generations, with topologies and parameters logged every 1500 generations. Results were evaluated using Tracer v1.6 [30]. The Effective Sample Size (ESS) values were > 200 for all parameters, suggesting the MCMC chains had sufficiently converged [40]. After discarding the first 25 % of generations as burn-in, the maximum clade credibility tree was constructed using TreeAnnotator 1.7.4 (available in the BEAST package), and then visualized with FigTree 1.3.1 [42].

### Morphological measurements

The following standard external measurements were taken from specimens collected in the field before their preparation using a millimeter ruler accurate to the nearest 0.5 mm: total length (TL), tail length (TAIL), hind foot length (HF) (not including claw), ear length (EAR) and forearm length (FA). Further, body mass (WT) was recorded in grams using a spring balance accurate to the nearest 0.5 g.

Cranial and dental measurements were obtained from cleaned skulls of voucher specimens using digital callipers accurate to the nearest 0.1 mm and following for the most part Freeman [43]: cranial — greatest skull length (SL), condyle-basal length (CBL), greatest zygomatic breadth (ZYGO), minimum interorbital width (IOW), greatest mastoid breadth (MAST), rostrum length (ROST), palatal length (PAL); and dental — total tooth row (C1-M3), upper molar row (UP MOL R), width at upper canines (C1-C1), width at upper posterior molars (M3-M3), height upper canine (UP CANIN), dentary length (DENT LEN), moment arm of temporal (MOM1 COR), total lower tooth row (I1-M3) and lower tooth row (LOWER TR). Only adult specimens were used in this study, as defined by the eruption of all permanent teeth (often showing some wear), the complete ossification of the basiosphenoid suture, and the development of the sagittal crest. All external and craniodental measurements used in the analyses were made by a single individual (SMG). The number of adult male *H. commersoni* available in the morphometric dataset was limited, and given there is evidence of sexual dimorphism in this species [16, 44], males were excluded from the morphometrics analyses. Intact skulls from 20 adult females were included in this study from 13 localities spanning the latitudinal distribution of *H. commersoni* in western Madagascar.

### Statistical analyses

Shapiro–Wilk’s test and Levene’s test were implemented to assess the assumptions of normality and equality of variances of variable characters in the dataset. Analysis of Variance (ANOVA) was carried out using post-hoc Tukey tests, to assess morphological and craniodental differences between the derived genetic clades.

Principal component analysis (PCA) was conducted separately on external and craniodental measurements to examine possible segregation of the different molecular clades, as well as geographic variation in *H. commersoni*. Further hierarchical cluster analysis was implemented using Ward’s method on both measurement data sets to provide additional confirmation of the factor loadings obtained and to identify natural groupings among samples (Tables 2 and 3) [45, 46]. Data were log-transformed to improve normality and homoscadisticity. All statistical analyses were carried out using SPSS (version 21.0, IBM SPSS Statistics).

**Table 2:** Reformed agglomeration table from hierarchical cluster analysis using Ward’s method of log-transformed external measurements of *Hipposideros commersoni*

No. of clusters	Agglomeration last step	Coefficients this step	Change
2	36.000	21.500	14.500
3	21.500	11.308	10.192
4	11.308	6.921	4.387
5	6.921	5.562	1.359
6	5.562	4.286	1.276

**Table 3:** Reformed agglomeration table from hierarchical cluster analysis using Ward’s method of log-transformed craniodental measurements of *Hipposideros commersoni*

No. of clusters	Agglomeration last step	Coefficients this step	Change
2	0.252	0.143	0.109
3	0.143	0.102	0.041
4	0.102	0.072	0.030
5	0.072	0.059	0.013
6	0.059	0.046	0.013

## Results

### DNA sequencing

The four genetic markers were successfully amplified for all 31 taxa included in the molecular portion of the study (Table 1). The aligned sequence data for each marker included (Table 1): CR, 481 bp (114 variable sites); *Cyt b*, 705 bp (60 variable sites); STAT, 476 bp (six variable sites); and OSTA5, 676 bp (nine variable sites). The nucleotide composition and the levels of variation of the two marker systems (mtDNA vs nucDNA) differed (Table 4). The mtDNA partition contained the highest number of variable characters (174 variable sites), while the ncDNA data was more conserved (15 variable sites). For the STAT gene, only eight unique haplotypes were identified. The haplotypic diversity for this dataset is high (h = 0.80), but the nucleotide diversity is low (π = 0.00274). For OSTA5 gene, 10 unique haplotypes were identified. Once again low levels of nucleotide variability were observed (π = 0.00264). As expected, CR contained the highest proportion of variable characters (24 % variable characters) followed by *Cyt b* (9 % variable characters).

**Table 4:** Characteristics of datasets used in this study

Gene	Total number of individuals	Total sites	Variable sites	Parsimony informative sites	Nucleotide frequencies			
					% A	% T	% C	% G
CR	31	481	114	72	32.73	27.24	25.74	14.29
*Cyt b*	31	705	60	38	26.90	27.06	30.86	15.17
bSTAT	31	476	6	2	20.17	27.91	28.77	23.14
OSTA5	31	676	9	6	23.85	25.46	27.04	23.65
Supermatrix	31	2336	189	118	25.87	26.84	28.26	19.03

Patterns of sequence variability are presented for two mtDNA regions (CR and *Cyt b*), two nuclear introns (bSTAT and OSTA5) and the combined data matrix. The total number of nucleotide sites, variable and parsimony informative sites, as well nucleotide frequencies are given for each partition and the combined data matrix

### Phylogenetic analysis

The phylogenetic analysis of each nuclear marker independently resulted in largely unresolved trees, which is not surprising given the few number of variable characters observed (Additional file 1: Figure S1 and S2; Table 4). The mtDNA marker topologies were better resolved (Additional file 1: Figure S3 and S4). There was no significant (ML bootstrap > 70 %; Bayesian posterior probability >95 %) conflict among the topologies recovered by the independent analysis of the four molecular markers [47] and the molecular data was concatenated (2336 bp, 118 variable characters). The ML and Bayesian analysis of the concatenated data matrix (mtDNA + ncDNA; Fig. 1) did not recover *H. commersoni* as a single monophyletic lineage. Two *H. commersoni* specimens (collected from the Isalo National Park, FMNH 175970, and from Itampolo, FMNH 184173) were placed in close association (ML bootstrap, 64; Bayes’ posterior probability, 1.00) to African *H. gigas* and *H. vittatus* (Clade A; Fig. 1). Clade A is genetically distant from the other Malagasy *H. commersoni* specimens. This level of divergence (2.6 % between Clades A and C to 3.1 % between Clades A and B [Table 5]) based on *Cyt b* uncorrected mean pairwise divergence is notable given that other *H. commersoni* specimens collected from these two localities cluster within Clade B (ML bootstrap, 99; Bayesian posterior probability, 1.00) together with specimens from localities in the southwest (Fig. 1). Clade C consists exclusively of specimens collected from the north. Clades B and C form a well-supported monophyletic lineage (ML bootstrap, 97; Bayes’ posterior probability, 1.00), sister to the lineage which includes Clade A, *H. gigas* and *H. vittatus* (ML bootstrap, 64; Bayes’ posterior probability, 1.00). These data strongly suggest the presence of several independently evolving lineages within *H. commersoni*. Clades B and C are geographically structured, with Clade C including specimens collected from northern Madagascar, while members of Clade B are more widely distributed in the south.

**Table 5:** Uncorrected mean pairwise distances based on analyses of the CR (below the diagonal) and *Cyt b* gene (above the diagonal) between major lineages of *Hipposideros commersoni* (Clades A, B, C) identified in the molecular analyses, *H. gigas* and *H. vittatus*

	Clade A	Clade B	Clade C	*H. gigas*	*H. vittatus*
Clade A		0.031	0.026	0.032	0.029
Clade B	0.072		0.019	0.031	0.028
Clade C	0.101	0.058		0.025	0.026
*H. gigas*	0.072	0.083	0.096		0.029
*H. vittatus*	0.074	0.077	0.090	0.069	

Uncorrected pairwise sequence distances for the two mtDNA regions (CR and *Cyt b*) are presented in Table 5. Genetically, Clade A is as distant from the *H. gigas*-*H. vittatus* species pair (respectively 3.2 % and 2.9 %) as it is from other *H. commersoni* placed in Clades B and C, again highlighting the uniqueness of this lineage.

### Molecular clock dating

**Fig. 2:**
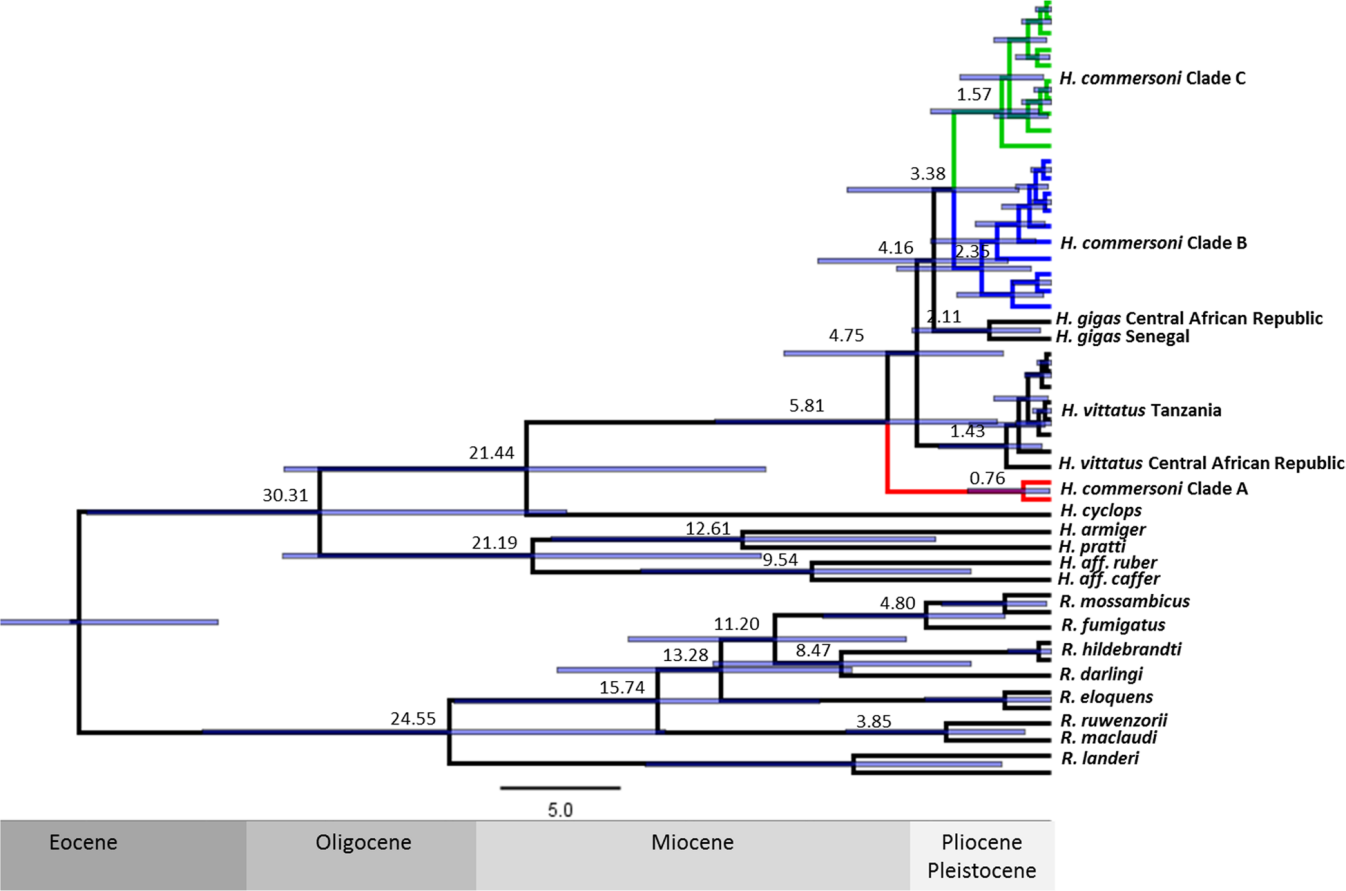
Maximum clade probability tree, inferred from the analysis of *Cyt b* data based on fossil calibration. *Values at nodes* indicate the posterior mean divergence dates in millions of years before present. *Shaded bars* indicate the 95 % highest posterior density (HPD) credibility intervals

The maximum clade probability tree (Fig. 2) inferred in BEAST supports the Garli and MrBayes phylogenies. Our analyses recovered *H. commersoni* Clade A as basal to all other members of the *H. commersoni* species group (*H. commersoni* Clades B & C, *H. gigas* and *H. vittatus*)*.* This suggests that *H. commersoni* Clades B and C are more closely related to the African taxa *H. gigas* and *H. vittatus* than to other Malagasy *H. commersoni* (Clade A). Molecular clock estimates using fossil calibration suggest that Clade A diverged from its sister taxa (*H. vittatus*, *H. gigas*, *H. commersoni* Clade B and C) during the Miocene (5.81 MYA; 95 % HPD 2.24–13.93). This divergence event is older than the separation of other established species groups, for example *Rhinolophus mossambicus* and *R. fumigatus*, which our molecular clock estimates suggests diverged 4.80 MYA (95 % HPD 1.90–9.45), and *R. ruwenzorii* and *R. maclaudi*, which diverged 3.86 MYA (95 % HPD 1.11–8.54) [37]. Clades B and C of the *H. commersoni* group last shared a common ancestor during the Pliocene (3.38 MYA; 95 % HPD 1.32–8.48, Fig. 2). The estimated divergence times using the substitution rate calibrated molecular clock resulted in more recent divergence dates (Additional file 2: Figure S5). For example, molecular clock estimates suggest that Clade A diverged from its sister taxa 4.20 MYA (95 % HPD 1.99–13.73) and the two sister Clades B & C shared their last common ancestor 2.55 MYA (95 % HPD 1.15–7.89, Table 6). The 95 % HPD intervals for divergence events from both analyses (fossil calibrated and substitution rate calibrated) were broad and but did not show considerable overlap. From Taylor et al. [37], *R. mossambicus* and *R. fumigatus*, diverged 6.96 MYA, which is older than our estimate and *R. ruwenzorii* and *R. maclaudi* about 2.99 MYA, which is more recent than our estimated. We suggest that using a calibration point allowed BEAST to estimate a more realistic clock rate. The substitution rate of 1.0 in the fossil calibrated clock allowed the determination of relative rates for each recovered clade.

**Table 6:** Divergence dates between major lineages of *Hipposideros commersoni* (Clades A, B, C) and *H. gigas* and *H. vittatus*

	Divergence times using fossil calibration point		Divergence times using fixed mean substitution rate	
Node:	Mean	95 % HPD (Mya)	Mean	95 % HPD (Mya)
Clade A	5.80	2.24–13.93	4.20	1.97–13.73
*H. vittatus*	4.75	2.01–11.09	3.68	1.80–10.88
*H. gigas*	4.16	1.77–9.68	3.17	1.50–9.00
Clade B & C.	3.38	1.32–8.48	2.55	1.15–7.99

Two molecular clock analyses were conducted. Fossil-calibrated values were estimated using a Bayesian lognormal relaxed-clock model, while the substitution rate calibrated values were estimated using the strict molecular clock model using a fixed mean substitution rate of 1.30 X 10^−8^ subs/site/year. The mean estimated values and the 95 % highest posterior density (HPD) ranges are given for the two molecular clocks. See Fig. 2 for the corresponding maximum clade probability trees

### Morphometrics

As Clade A was only comprised of two individuals, statistical comparisons were only made between animals belonging to Clades B and C. Shapiro–Wilk’s test (*P* > 0.05) and a visual inspection of their histograms showed that the variable characters in the dataset were normally distributed. Levene’s test verified the equality of variances in the samples (*P* > 0.05).

Analysis of variance (ANOVA) revealed significant variation in four of six external variables and 15 of 16 craniodental variables when taxa were sorted into Clades A, B or C following the results of molecular analyses (Table 7). Clade A is morphologically similar to Clade B, but is morphologically differentiated from Clade C (Table 7). The larger Clade C bats had significantly greater total length, tail length, ear length and forearm length than specimens assigned to Clade B, yet there were no significant differences in hindfoot length and body mass between the clades (Table 7). In parallel, craniodental measurements were significantly larger in Clade C than Clade B, except minimum interorbital width (Table 7).

**Table 7:** Summary of body mass, external body and craniodental measurements, and results of one-way ANOVAs and Tukey *post hoc* tests for *Hipposideros commersoni* based on the molecular clades defined in this study

Variable		Clade A	Clade C	Clade B	One-way ANOVA		Post hoc Tukey tests
					F_(2, 16)_	P	
TL					6.50	0.01	*P* = 0.03;
							Clade B < C
	N	2	9	8			
	Mean	121.0	133.6	125.6			
	Std. Deviation	-	4.50	7.23			
	Minimum	119	125	115			
	Maximum	123	138	137			
TAIL					7.03	0.006	*P* = 0.006;
							Clade B < C
	N	2	9	8			
	Mean	32	36.8	31.0			
	Std. Deviation	-	2.91	3.66			
	Minimum	30	31	25			
	Maximum	34	41	35			
HF					1.51	ns	ns
	N	2	9	8			
	Mean	14.5	15.8	14.8			
	Std. Deviation	0.71	0.44	1.98			
	Minimum	14	15	13			
	Maximum	15	16	18			
EAR					10.16	0.01	*P* = 0.02;
							Clade B < C
	N	2	9	8			
	Mean	26.5	30.7	28.6			
	Std. Deviation	-	0.71	1.85			
	Minimum	26	30	26			
	Maximum	27	32	31			
FA					6.41	0.009	*P* = 0.02;
							Clade B < C
	N	2	9	8			
	Mean	80.5	86.4	82.3			
	Std. Deviation	-	2.88	2.96			
	Minimum	80	82	79			
	Maximum	81	91	87			
WT					0.20	ns	ns
	N	2	9	8			
	Mean	40.5	42.11	39.69			
	Std. Deviation	-	5.07	7.40			
	Minimum	26	30	29			
	Maximum	55	47	49			
					F_(2, 14)_	P	
SL					9.82	0.002	*P* = 0.011;
							Clade B < C
	N	2	8	7			
	Mean	26.95	29.72	28.08			
	Std. Deviation	-	0.67	1.20			
	Minimum	26.5	28.2	26.6			
	Maximum	27.4	30.5	29.9			
CBL					9.68	0.002	*P* = 0.009;
							Clade B < C
	N	2	8	7			
	Mean	23.9	26.34	24.78			
	Std. Deviation	-	0.60	1.12			
	Minimum	23.6	25.1	23.4			
	Maximum	24.2	27.1	26.6			
ZYGO			6.32	0.01	ns		
	N	2	8	7			
	Mean	14.1	15.65	14.83			
	Std. Deviation	-	0.59	0.64			
	Minimum	13.6	15	14.1			
	Maximum	14.6	16.8	15.8			
IOW					0.36	ns	ns
	N	2	8	7			
	Mean	2.9	3.01	2.93			
	Std. Deviation	-	0.13	0.26			
	Minimum	2.6	2.8	2.6			
	Maximum	3.2	3.2	3.3			
MAST			5.91	0.034			*P* = 0.014;
							Clade B < C
	N	2	8	7			
	Mean	12.2	13.67	12.91			
	Std. Deviation	-	0.51	0.69			
	Minimum	11.7	12.9	12.2			
	Maximum	12.7	14.4	13.9			
ROST					4.34	0.034	*P* = 0.038;
							Clade B < C
	N	2	8	7			
	Mean	10.95	11.70	10.94			
	Std. Deviation	-	0.38	0.68			
	Minimum	10.7	11	10.1			
	Maximum	11.2	12.1	12			
PAL					4.34	0.028	ns
	N	2	8	7			
	Mean	3.65	4.47	4.07			
	Std. Deviation	-	0.27	0.43			
	Minimum	3.2	4	3.7			
	Maximum	4.1	4.8	4.9			
C1-M3					10.97	0.001	*P* = 0.004;
							Clade B < C
	N	2	8	7			
	Mean	9.45	10.55	9.79			
	Std. Deviation	-	0.26	0.50			
	Minimum	9.3	10	9.3			
	Maximum	9.6	10.8	10.8			
UP MOL R					9.79	0.002	*P* = 0.003;
							Clade B < C
	N	2	8	7			
	Mean	7.35	7.87	7.36			
	Std. Deviation	-	0.17	0.31			
	Minimum	7.3	7.6	7			
	Maximum	7.4	8.2	8			
C1-C1					11.69	0.001	*P* = 0.002;
							Clade B < C
	N	2	8	7			
	Mean	6.90	8.16	7.17			
	Std. Deviation	-	0.46	0.45			
	Minimum	6.6	7.3	6.7			
	Maximum	7.2	8.7	7.9			
M3-M3					7.33	0.007	*P* = 0.011;
							Clade B < C
	N	2	8	7			
	Mean	9.85	10.82	10.03			
	Std. Deviation	-	0.24	0.64			
	Minimum	9.7	10.4	9.2			
	Maximum	10	11.1	11			
UP CANIN					4.50	0.031	ns
	N	2	8	7			
	Mean	4.35	5.09	4.60			
	Std. Deviation	-	0.26	0.45			
	Minimum	3.9	4.7	3.8			
	Maximum	4.8	5.4	5.3			
DENT LEN					8.46	0.004	*P* = 0.011;
							Clade B < C
	N	2	8	7			
	Mean	17.75	19.57	18.33			
	Std. Deviation	-	0.48	0.95			
	Minimum	17.6	18.5	17.1			
	Maximum	17.9	20	19.6			
MOM1 COR					4.01	0.042	ns
	N	2	8	7			
	Mean	5.65	6.22	5.76			
	Std. Deviation	-	0.21	0.49			
	Minimum	5.5	6.0	5.0			
	Maximum	5.8	6.6	6.4			
I1-M3					7.78	0.005	*P* = 0.014;
							Clade B < C
	N	2	8	7			
	Mean	11.75	12.99	12.13			
	Std. Deviation	-	0.30	0.70			
	Minimum	11.6	12.5	11.1			
	Maximum	11.9	13.3	13.2			
LOWER TR					11.20	0.001	*P* = 0.003;
							Clade B < C
	N	2	8	7			
	Mean	10.70	11.87	10.97			
	Std. Deviation	-	0.27	0.57			
	Minimum	10.5	11.5	10.2			
	Maximum	10.9	12.2	11.9			

See materials and methods for definitions of variable acronyms. ns = not significant

**Fig. 3:**
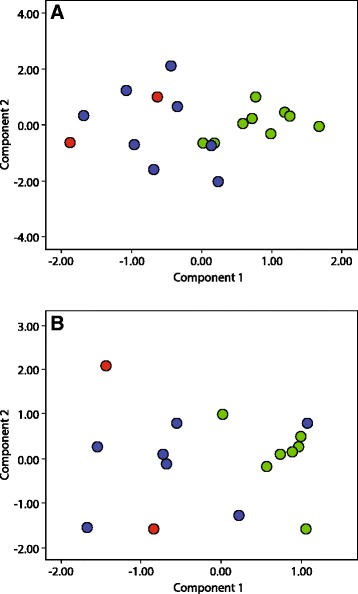
Principal component analyses of female *Hipposideros commersoni* for **a** log-transformed external variables for 19 specimens and **b** log-transformed craniodental variables for 17 specimens (*red* = Clade A, *blue* = Clade B, *green* = Clade C)

The first two unrotated principal components (PC1 and PC2) explained 67.6 % of the total variance in external measurements (Fig. 3a) and 88.2 % of total variance in cranial-dental morphology (Fig. 3b). PCA plots of external and craniodental variables recovered Clades B and C as two distinct groups with little overlap. In contrast, individuals of Clades A and B overlapped in morphological variables. Because several of the external morphology variables and most of the craniodental variables loaded high on PC1, we interpreted this component as a proxy for size. Both sets of variables suggest that *H. commersoni* Clades A and B are smaller than those from Clade C (Fig. 3). In the case of external measurements, PC2 showed an inverse relationship between tail length and hind foot length – bats that had high loadings on PC2 had a relatively long tail but short hind foot, whereas bats that had low loadings on PC2 had a relatively short tail but long hind foot (Table 8). In the case of craniodental variables, PC2 indicated skull robustness (Table 9) - bats that loaded high on PC2 had a relatively larger interorbital width than bats that loaded low on PC2.

**Table 8:** Character loading for the first three components (PC) in a Principal Component analysis based on external measurements of *Hipposideros commersoni*

Variable	PC1	PC2	PC3
TAIL	0.543	0.566	0.518
HF	0.541	−0.657	0.263
EAR	0.850	−0.197	−0.185
FA	0.824	−0.317	−0.172
TL	0.774	0.308	0.265
WT	0.519	0.461	−0.636

**Table 9:** Character loading for the first three components (PC) in a principal component analysis based on craniodental measurements of *Hipposideros commersoni*

Variable	PC1	PC2	PC3
SL	0.980	−0.140	−0.026
CBL	0.980	−0.110	−0.034
ZYGO	0.898	−0.288	0.171
IOW	0.396	0.805	0.315
MAST	0.909	−0.264	0.048
ROST	0.800	0.051	0.446
PAL	0.865	−0.315	0.019
C1-M3	0.972	0.039	−0.071
UP MOL R	0.869	0.258	0.021
C1-C1	0.925	−0.222	−0.129
M3-M3	0.976	0.034	0.010
UP CANIN	0.688	0.578	−0.342
DENT LEN	0.977	−0.038	−0.082
MOM1 COR	0.908	−0.035	0.200
I1-M3	0.951	0.137	−0.210
LOWER TR	0.960	0.114	−0.149

**Fig. 4:**
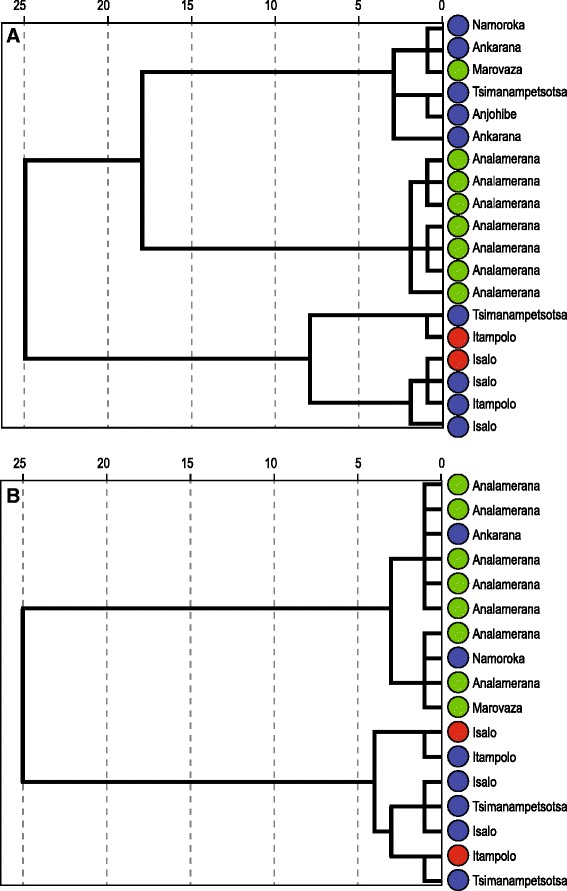
Hierarchical clustering dendrogram for **a** log-transformed external variables for 19 specimens and **b** log-transformed craniodental variables of 17 female *Hipposideros commersoni*. Collection locality information is assigned to each individual and color-coding is based on main lineages recovered by molecular data

Two major clusters were recovered from the dendrograms produced by the hierarchical cluster analyses of external and craniodental variables, supporting the PCA analysis. The first cluster, recovered in both dendrograms, included all eight individuals from northern Madagascar assigned to Clade C and two animals genetically assigned to Clade B (FMNH 221308 from Ankarana and FMNH 175777 from Namoroka). The second cluster contained the smaller southern individuals from Clades A and B (Fig. 4), which confirms that the two genetically divergent animals belonging to Clade A are morphologically similar to animals from Clade B.

## Discussion

This study combines evidence from molecular (mtDNA and ncDNA) and morphological characters to provide support for the reciprocal monophyly of several independently evolving lineages within *Hipposideros commersoni*, occurring on Africa and nearby islands, as well on Madagascar. One of the principal questions addressed is the evolutionary history and systematic relationships of Malagasy populations currently assigned to *H. commersoni*, as well as African populations currently placed within the *H. commersoni* species group [1].

The results suggest that previous taxonomic treatments of the group underestimated species diversity of *H. commersoni* and that a cryptic species appears to be present. Although our geographic sampling did not cover the complete range of this species, specifically the eastern portion of the island, the results indicate non-monophyly with respect to Madagascar of different recovered Malagasy clades.

Single locus or multilocus molecular data and/or morphological differences have been used previously to identify cryptic species diversity in southeast Asian [10, 48, 49] and African [50, 51] hipposiderids. Based on an analysis of the *H. larvatus* species complex using mitochondrial control region markers, morphology and bioacoustics two forms were identified (*H. khasiana* and *H. grandis*) that were differentiated based on haplotypic structure and phonics [52]. In another example, *H. khaokhouaensis* from Laos is similar in general body size and shape to its sister species *H. rotalis*, but differs in aspects of the noseleaf, skull structure related to bioacoustics and echolocation frequency [4]. The phylogenetic relationships within the African *H. ruber* species complex were investigated [51] using *Cyt b* to determine the taxonomic status of two divergent genetic forms often found in sympatry in Senegal, which might represent cryptic species despite being morphologically indistinguishable. However, in this latter case, absence of nuclear gene flow between these two reputed forms remains to be investigated to demonstrate their reproductive isolation.

All *H. commersoni* sequenced in the current study were from the western half or extreme north of Madagascar. The molecular analyses presented herein indicate that *H. commersoni* as currently diagnosed is not monophyletic with respect to Madagascar, and with strong support for the presence of divergent lineages. As two individuals from Isalo (FMNH 175970) and Itampolo (FMNH 184173) form a well-supported monophyletic group (Clade A), basal to African *H. vittatus* and *H. gigas*, and separate from the balance of Malagasy *H. commersoni* (Clades B and C), a single origin of this species complex on the island is not supported. The long branches separating these clades (ranging from 2.6 to 3.2 % uncorrected sequence divergence) indicate relatively deep independent evolutionary trajectories of several million years based on molecular clock inferences. Although Clade A was only significantly supported by the mitochondrial data, this is not surprising, given the relatively conservative nature of the nuclear markers sequenced. The absence of haplotype sharing in OSTA5 gene for Clades B and C, however, does indicate at least some degree of genetic isolation between these two groups. These results indicate that members of the *H. commersoni* species group do not represent a single widespread Afro-Malagasy taxon. Furthermore, the molecular data supports a certain level of divergence between Clades B (in the north) and C (in the south). Additional samples are needed to ascertain whether the recovered phylogeny is an artefact of sampling or the result of isolation by distance. The sequence divergence of Clade A with respect to the balance of *H. commersoni* (Clades B and C) is comparable with that observed between African *H. vittatus* and *H. gigas*, and based on the molecular clock analysis, it is estimated that the Clade A lineage diverged from its sister taxa during the Miocene.

Genetic divergence in mitochondrial genes varies widely among species. Avise [53] highlighted that due to the matrilineal nature of inheritance of mitochondrial genes, relatively deep divergences do not necessarily correspond to species boundaries. Further, significant nuclear gene flow may occur among divergent mitochondrial phylogroups. Using the published literature, Baker and Bradley [54] found an interval of 3.3–14.7 % uncorrected genetic distance between sister species of bats, and distances ranging from 0.6–2.3 % encompassing intraspecific variation. These values have been corroborated by recent studies of cryptic species of Asian *Hipposideros*, which show three different levels of interspecific divergences: 1) as low as 3.9 %, with supporting evidence from external and craniodental morphology, as well as bioacoustics [4]; 2) an intermediate level of 6.5 %, with corroborating evidence from bioacoustics [55]; and 3) as high as 13.4 %, with corroborating evidence from bioacoustics [52]. The sequence divergence values recovered in the current study separating Clade A from other *H. commersoni* (Clades B and C) suggest that previous taxonomic conclusions underestimated the species diversity of Malagasy bats currently classified as *H. commersoni*.

Within the portion of the phylogeny composed of most individuals assigned to *H. commersoni*, the molecular data support two largely geographically non-overlapping clades: a northern group (Clade C) with a relatively limited range and a southern group with a broader geographical distribution (Clade B). The molecular clock analyses indicate that these two clades diverged from one another approximately 3.38 MYA. Morphometric analyses are generally consistent with the molecular data, suggesting a north–south break between animals assigned to Clades B and C. The exception was in Ankarana (far north), where the two lineages co-occur but individuals from each clade could not be differentiated based on multivariate analyses of external and craniodental measurements (Fig. 4). Patterns of morphological variation were not uniform or falling along well-defined clines, such as latitude, and members of these two clades do not completely separate from one another. In term of genetics, based on currently available samples, Clades B and Clade C are differentiated, for example, the uncorrected *Cyt b* sequence divergence is 1.9 %. In the case of the two individuals falling within Clade A, they are genetically distinct from those in Clade B, but show no apparent morphological differentiation. Hence, we interpret this variation as some form of incipient speciation between animals assigned to Clades B and C.

Ramasindrazana et al. [44] have recently analyzed echolocation calls of animals referred to as *H. commersoni* captured in western Madagascar. They found latitudinal variation - animals from the north being larger and emitting lower call frequencies and those from the south smaller and emitting higher call frequencies. On average, females, referred to *H. commersoni*, from the north (Ankarana) deviate from the allometric relationship with lower resting frequency of echolocation calls than predicted from body size. These authors suggested that this pattern might be explained by either regional variation in bioacoustics, intra-island migratory movements or the presence of a cryptic species. The animals that deviated from the pattern were not sequenced in this current study and no further interpretation can be offered.

### Evolution of Malagasy *Hipposideros*

A particularly striking result of the current analyses is the existence of a previously unrecognized clade of Malagasy *H. commersoni* (Clade A), estimated to have diverged from sister taxa (Malagasy *H. commersoni* Clades B and C, and African *H. gigas* and *H. vittatus*) during the late Miocene (5.81 MYA). *Hipposideros gigas and H. vittatus* are more closely related to *H. commersoni* Clades B and C, suggestive of two dispersal hypotheses. The first scenario is that Clade A and Clade B-C originated from two independent African mainland-to-Madagascar dispersal events, with Clade A arriving on the island during the Miocene (approximately 5.8 MYA) and Clade B-C more recently (approximately 3.38 MYA). A second hypothesis is that the *H. commersoni* group evolved on Madagascar and at some point after the end of the Miocene, a population related to Clade A crossed the Mozambique Channel and colonized the African continent leading to two recognized extant forms, *H. vittatus* and *H. gigas*, that are morphologically and karyologically similar [17, 36]. Following this second hypothesis, speciation took place within the Malagasy population, giving rise to Clades B and C representing the most recent branch of this lineage.

Madagascar was cooler and drier during periods of Pleistocene glaciation, which lead to habitat shifts and forced some taxa to retreat into refugia [56–58], in different high mountain areas [59]. Expansion from refugia would have occurred during warmer periods. *Hipposideros* bones from relatively recent geological deposits are known from several sites on the island [60]. Subfossils from Tsimanampetsotsa, extreme southwest, identified as *Hipposideros* were slightly smaller than typical *H. commersoni* [61], which occur in this region today [11]. Samonds [62] conducted research on *Hipposideros* subfossils from Anjohibe Cave in the northwest of Madagascar, and the excavated fossils were dated between 10,000 and 80,000 years ago. Samonds [62] identified three morphological forms of *Hipposideros* from these deposits: 1) those fitting with extant *H. commersoni*; 2), *H. besaoka*, which was described as a new species, being larger and more robust than *H. commersoni*; and 3), *Hipposideros* sp. cf. *H. commersoni*, which appeared to have some dental differences from modern *H. commersoni. Hipposideros besaoka* and *H. commersoni* were sympatric and presumably living in the Anjohibe Cave during the same period, and they show a small amount of overlap in some dental measurements, not related to sexual dimorphism [62]. Our morphological and molecular data suggests parallel results in modern populations of *H. commersoni*, with Clade B and C known to occur in sympatry at one northern locality. This raises the intriguing possibility that one of the phylogenetic clades identified in this paper (Clade B or Clade C), might be referable to *H. besaoka* and, in this case, this species is not extinct. Further fine-scale phylogeographic studies using variable nuclear markers such as microsatellites are needed to clarify species boundaries and give a greater understanding of the processes underpinning the evolution of these taxa across Madagascar.

### Geographically correlated population structure

Within *H. commersoni* (Clade B-C), the molecular data support two regionally associated clades: a small-bodied southern group with a broad geographical distribution (Clade B) and a large-bodied northern group (Clade C) with a relatively limited range. The molecular clock analyses indicate that these two clades diverged from one another approximately 3.38 MYA. Morphometric data are consistent with the molecular data, suggesting a north–south break in distribution. These two lineages are not completely allopatric. In Ankarana, sequenced individuals assigned to these two genetic clades could not be distinguished using external and craniodental measurements (Fig. 3). The morphometric data in the present study is consistent with conclusions of a previous study on geographic variation in morphology of this taxon in western Madagascar [16]. Specimens grouped into two distinct morphotypes, a larger morphotype found in northern Madagascar (from Analamerana to Ankarana and south to Bemaraha) and a smaller morphotype widely distributed in the south, from Isalo to Tsimanampetsotsa. Ranivo and Goodman [16] found that male *H. commersoni* do not show the same pattern and are largely homogenous in size across these zones.

At least three other Malagasy bat species, *Paratriaenops furculus* [63], *Chaerephon leucogaster* [64] and *Myotis goudoti* [65] show similar haplotypic segregation along a latitudinal gradient. However, the latitudinal distribution of different clades and the calculated expansion periods of the other species differ from late Pleistocene in *M. goudoti* to early Holocene in *C. leucogaster*, suggesting that no common historical process underlies the different demographic events between these taxa [64, 65].

Ranivo and Goodman [16] found both *H. commersoni* morphotypes in Isalo. The morphologically divergent animals from Isalo included two specimens (FMNH 175973 and 175975) that were collected on the same day and at the same cave site as the Isalo specimen (FMNH 175970) analyzed in our molecular study, which falls into Clade A. This latter specimen morphologically aligns with the smaller southern individuals, while FMNH 175973 and 175975 are of the larger northern morphotype. This may indicate some form of intra-island movements.

In eastern Africa, seasonal fluctuations in abundance of prey utilized by large hipposiderids are pronounced, which can result in food shortages during the cool dry season. These shifts in the resource base have been invoked to explain local seasonal movement in *H. vittatus*/*H. gigas* to areas with greater food abundance [7, 66]. It is unclear if *H. commersoni* remains inactive in caves during times of resource shortage or if local populations migrate to other sites. Large hipposiderid bats have high wing loading and low to medium aspect ratios [67], which may favor relatively quick, long-distance movements, allowing certain populations to track food resources [68, 69]. The colonization and speciation history of *H. commersoni* on Madagascar, as represented by a single species occurring on the island, is certainly more complex than currently understood. Further studies including increased spatial sampling and the use of additional molecular markers particularly faster evolving nuclear markers are needed to fully resolve the evolutionary history and associated systematics of the different clades occurring on Madagascar.

## Conclusions

This study provides evidence, particularly from mitochondrial data, for the existence of at least two sympatrically-occurring species of the genus *Hipposideros* on Madagascar. Absence of nuclear gene flow between groups remains to be established to verify their reproductive isolation, yet the lack of haplotype sharing in OSTA5 for Clades B and C indicates some degree of genetic isolation between these clades. Subfossil evidence indicates that in the recent geological past two species, *H. commersoni* and the presumed extinct *H. besaoka*, occurred in sympatry [62]. Given that we have recovered two genetically distinct lineages of *H. commersoni* (Clades B and C) living on occasion in sympatry, this might indicate that one of them is *H. besaoka* and, hence, still extant. A detailed morphological comparison of the type series of Samonds’ [62] *H. besaoka* with modern *H. commersoni* represented in our data is needed to test this intriguing possibility and crucial before the description of a possible undescribed species.

## Appendix 1

**Table 10:** Collection details of specimens of *Hipposideros commersoni* included in analyses of external and cranio-dental morphometry and and molecular variation

Museum number	Province/Locality (numbers in parentheses refer to those shown in Fig. 1)	Clade (from Fig. 2)	Latitude	Longitude	Collection date	Sex	Alt. (m)
FMNH 169707	Antsiranana/RS d’Ankarana, 2.6 km E Andrafiabe, near Andrafiabe Cave	C	49.0567	−12.9317	November 2001	Female	50
FMNH 175777	Mahajanga/RNI de Namoroka, near source of Mandevy River, 32 km NW Andranomavo	B	45.345	−16.38	October 2002	Female	100
FMNH 175966	Fianarantsoa/just outside PN de l’Isalo, along Menamaty River, 8 km N Ranohira (RN7)	B	45.3917	−22.4856	December 2002	Female	700
FMNH 175970	Fianarantsoa/PN de l’Isalo, along Sahanafa River, 28 km SE Berenty-Betsileo	A	45.2933	−22.3167	December 2002	Female	550
FMNH 176155	Toliara/Forêt des Mikea, 9.5 km W Ankiloaka	B	43.5233	−22.7783	February 2003	Male	80
FMNH 177302	Mahajanga/SF d’Ampirojoa	B	46.81	−16.315	April 2003	Male	100
FMNH 178806	Antsiranana/RS d’Analamerana, Grotte de Bazaribe, 3.6 km SE Menagisy	C	49.47352	−12.7121	January 2004	Female	90
FMNH 178808	Antsiranana/RS d’Analamerana, Grotte de Bazaribe, 3.6 km SE Menagisy	C	49.47352	−12.7121	January 2004	Female	90
FMNH 178809	Antsiranana/RS d’Analamerana, Grotte de Bazaribe, 3.6 km SE Menagisy	C	49.47352	−12.7121	January 2004	Female	90
FMNH 178810	Antsiranana/RS d’Analamerana, Grotte de Bazaribe, 3.6 km SE Menagisy	C	49.47352	−12.7121	January 2004	Female	90
FMNH 178811	Antsiranana/RS d’Analamerana, Grotte de Bazaribe, 3.6 km SE Menagisy	C	49.47352	−12.7121	January 2004	Female	90
FMNH 178815	Antsiranana/RS d’Analamerana, Grotte de Bazaribe, 3.6 km SE Menagisy	C	49.47352	−12.7121	January 2004	Female	90
FMNH 183932	Toliara/PN de Tsimanampetsotsa, 6.5 km NE Efoetse, near Mitoho Cave	B	43.75	−24.05	October 2004	Female	50
FMNH 183934	Toliara/PN de Tsimanampetsotsa, 6.5 km NE Efoetse, near Mitoho Cave	B	43.75	−24.05	October 2004	Female	50
FMNH 184170	Toliara/Grotte d’Androimpano, 4.2 km NE Itampolo (village), on old road to Ejeda	B	43.96328	−24.6502	February 2005	Female	110
FMNH 184173	Toliara/Grotte d’Androimpano, 4.2 km NE Itampolo (village), on old road to Ejeda	A	43.96328	−24.6502	February 2005	Female	110
FMNH 184030	Mahajanga/4.2 km SE Marovaza, in cave	C	47.30797	−14.966	April 2005	Female	40
FMNH 183980	Antsiranana/Montagne de Français, Forêt d’Ampitiliantsambo	C	49.38453	−12.3371	January 2005	Female	210
FMNH 217940	Fianarantsoa/PN de l’Isalo, 10.5 km SW Ranohira, Hotel Jardin du Roi	B	45.29	−22.31	December 2011	Female	
UADBA 32987	Antsiranana/PN d’Ankarana, 2.6 km E Andrafiabe, in forest near AndrafiabeCave	C	49.05667	−13.93167	September 2012	Female	50
FMNH 221308	Antsiranana/PN d’Ankarana, 2.6 km E Andrafiabe, in forest near Andrafiabe Cave	B	49.05667	−13.93167	September 2012	Female	50
UADBA 32916	Mahajanga/Grotte d’Anjohibe, 3.7 km NE Antanamarina	B	46.88598	−15.53815	September 2012	Female	100

All listed specimens were included in the molecular analyses. and with the exception of those underlined, were also included in the morphometric craniodental analysis. For voucher specimens used in molecular analyses, clade assignments are given based on the supermatrix data presented in Fig. 2. Localities correspond with those presented in Fig. 1. Institutional acronyms are as follows: UADBA: Université d’Antananarivo, Département de Biologie Animale, Antananarivo; FMNH: Field Museum of Natural History, Chicago. Site acronyms: *PN* Parc National, *RNI* Réserve Naturelle Intégrale, *RS* Réserve Spéciale, *SF* Station Forestière

## Additional files

Additional file 1: Figures S1 to S4. Single-gene trees. Maximum likelihood tree inferred from CR (S1) , *Cyt b* (S2), bSTAT (S3) and OSTA5 (S4). Posterior probability values and maximum likelihood bootstrap support (in that order) are shown at the nodes. S1) Maximum likelihood tree inferred from mtDNA control region data. Bayesian posterior probability values and maximum likelihood bootstrap support (in that order) are shown at the nodes. S2) Maximum likelihood tree inferred from mtDNA *Cyt b data*. Posterior probability values and maximum likelihood bootstrap support (in that order) are shown at the nodes. S3) Maximum likelihood tree inferred from nuclear intron bSTAT. Posterior probability values and maximum likelihood bootstrap support (in that order) are shown at the nodes. S4) Maximum likelihood tree inferred from the nuclear intron OSTA5. Posterior probability values and maximum likelihood bootstrap support (in that order) are shown at the nodes. (DOC 21377 kb) Additional file 2: Figure S5. Alternative maximum clade probability tree, inferred from the analysis of *Cyt b* data. A strict molecular clock model with a fixed mean substitution rate of 1.30 × 10^-8^ subs/site/year was performed. Values at nodes indicate the posterior mean substitution rate (subs/site/year). Shaded bars indicate the 95% highest posterior density (HPD) credibility intervals. (DOC 118 kb)
