# Supplementary material for: Cryptic diversity in Hipposideros commersoni sensu stricto (Chiroptera: Hipposideridae) in the western portion of Madagascar
Source: BMC Evol Biol. 2015 Oct 30;15:235. doi: 10.1186/s12862-015-0510-2 (PMC4628396; doi:10.1186/s12862-015-0510-2)
Supplement: Additional file 1: Figures S1 to S4. — Single-gene trees. Maximum likelihood tree inferred from CR (S1) , Cyt b (S2), bSTAT (S3) and OSTA5 (S4). Posterior probability values and maximum likelihood bootstrap support (in that order) are shown at the nodes. S1) Maximum likelihood tree inferred from mtDNA control region data. Bayesian posterior probability values and maximum likelihood bootstrap support (in that order) are shown at the nodes. S2) Maximum likelihood tree inferred from mtDNA Cyt b data. Posterior probability values and maximum likelihood bootstrap support (in that order) are shown at the nodes. S3) Maximum likelihood tree inferred from nuclear intron bSTAT. Posterior probability values and maximum likelihood bootstrap support (in that order) are shown at the nodes. S4) Maximum likelihood tree inferred from the nuclear intron OSTA5. Posterior probability values and maximum likelihood bootstrap support (in that order) are shown at the nodes. (DOC 21377 kb) [file 12862_2015_510_MOESM1_ESM.doc]

**Additional file 1. Figures S1 to S4. Single-gene trees.** Maximum likelihood tree inferred from CR (S1) , *Cyt b* (S2), bSTAT (S3) and OSTA5 (S4). Posterior probability values and maximum likelihood bootstrap support (in that order) are shown at the nodes.


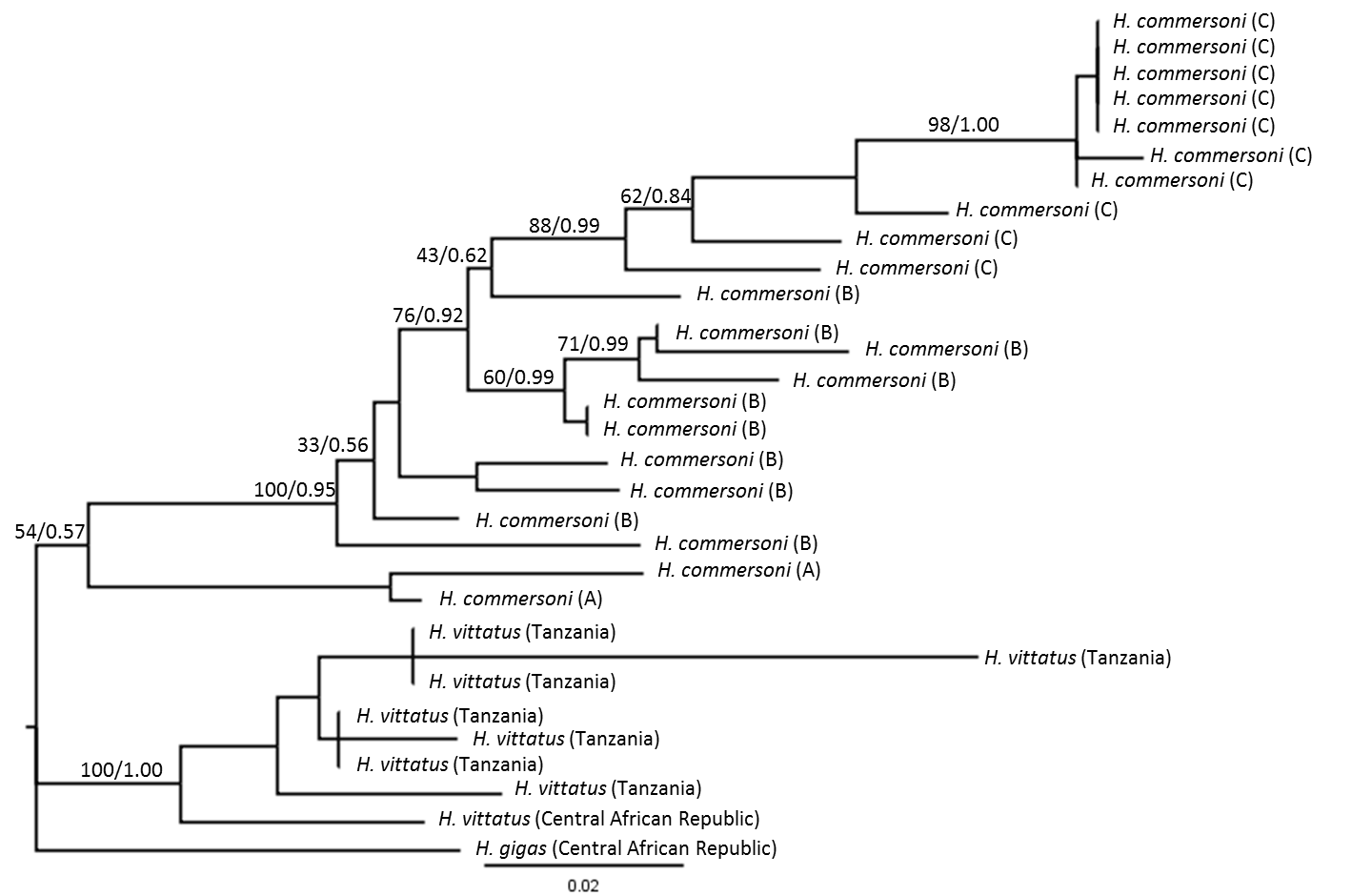


S1) Maximum likelihood tree inferred from mtDNA control region data. Bayesian posterior probability values and maximum likelihood bootstrap support (in that order) are shown at the nodes.


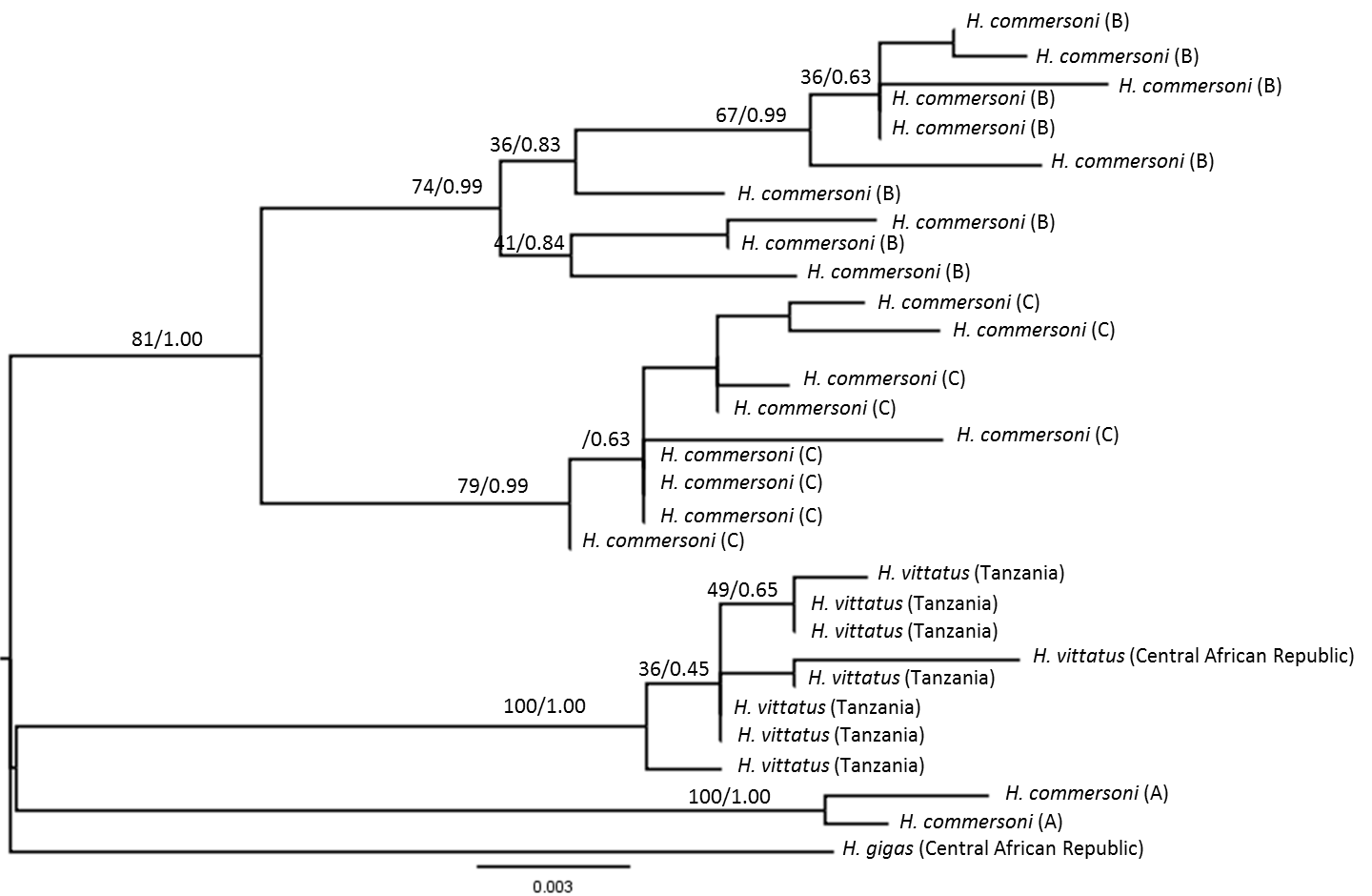


S2) Maximum likelihood tree inferred from mtDNA *Cyt b data*. Posterior probability values and maximum likelihood bootstrap support (in that order) are shown at the nodes.


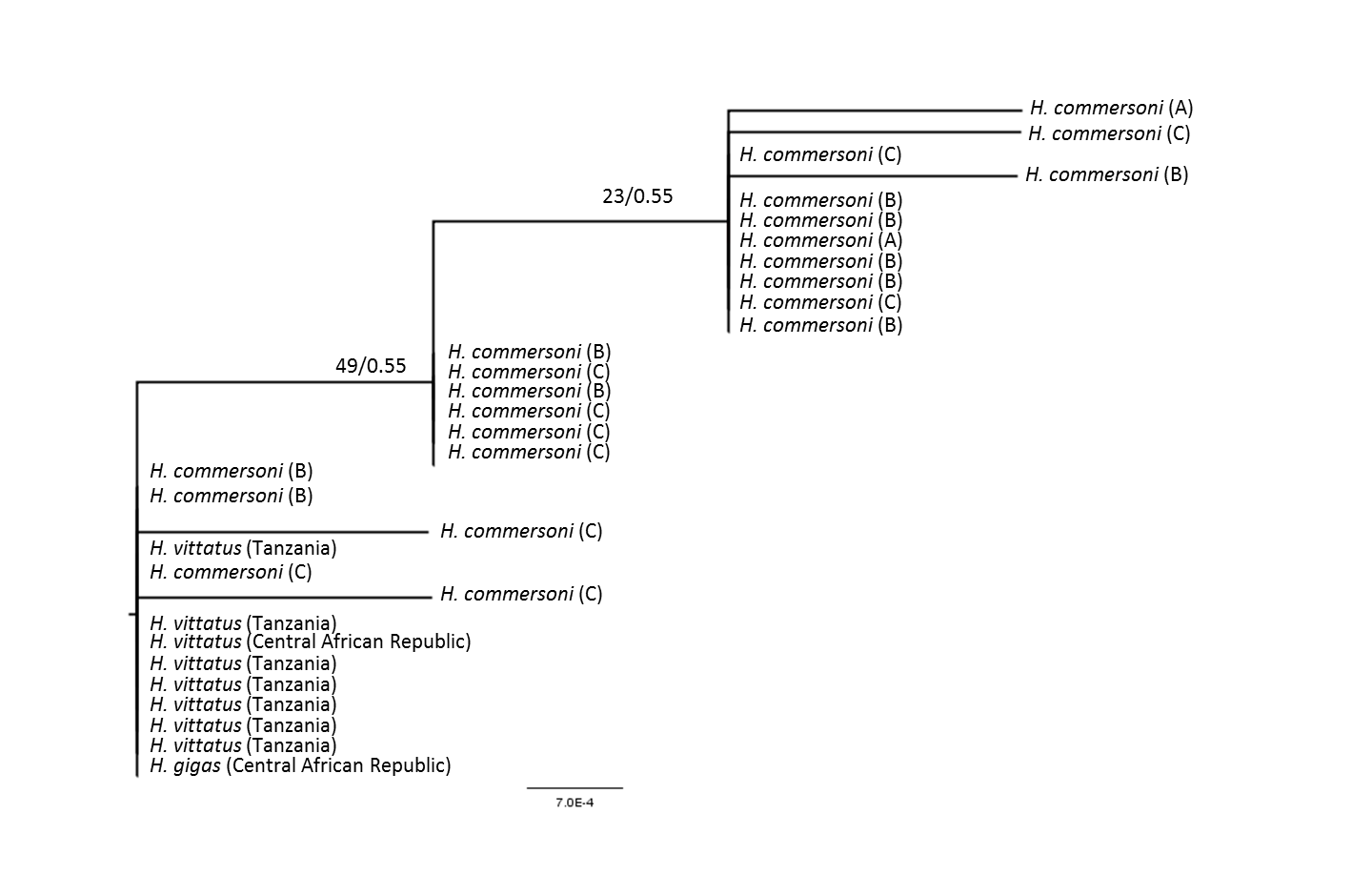


S3) Maximum likelihood tree inferred from nuclear intron bSTAT. Posterior probability values and maximum likelihood bootstrap support (in that order) are shown at the nodes.


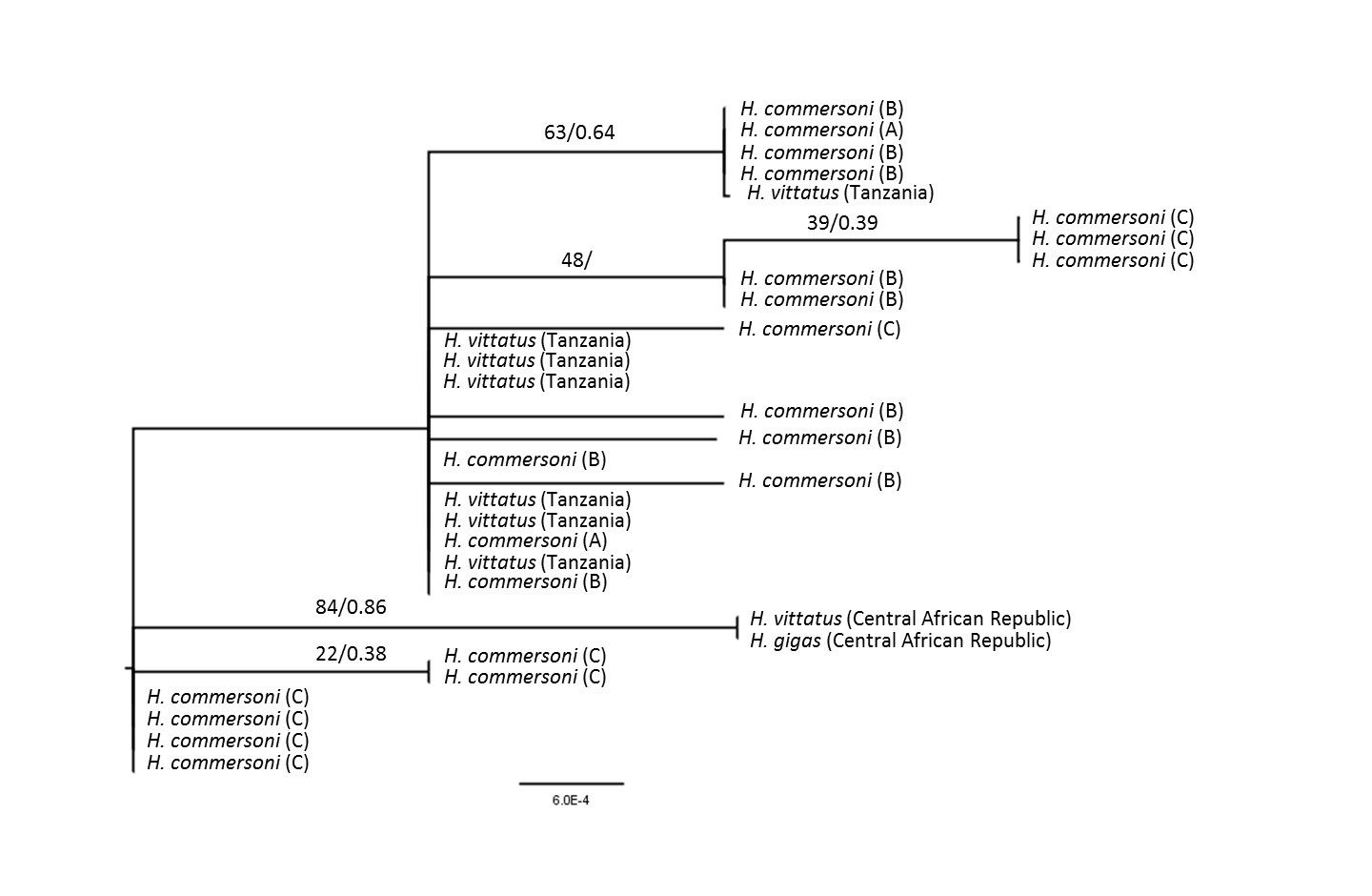


S4) Maximum likelihood tree inferred from the nuclear intron OSTA5. Posterior probability values and maximum likelihood bootstrap support (in that order) are shown at the nodes.
