# Supplementary material for: Cryptic diversity in Hipposideros commersoni sensu stricto (Chiroptera: Hipposideridae) in the western portion of Madagascar
Source: BMC Evol Biol. 2015 Oct 30;15:235. doi: 10.1186/s12862-015-0510-2 (PMC4628396; doi:10.1186/s12862-015-0510-2)

**Additional file 2. Figure S5 Alternative maximum clade probability tree, inferred from the analysis of *Cyt* *b* data**. A strict molecular clock model with a fixed mean substitution rate of 1.30 X 10^-8^ subs/site/year was performed. Values at nodes indicate the posterior mean substitution rate (subs/site/year). Shaded bars indicate the 95% highest posterior density (HPD) credibility intervals.
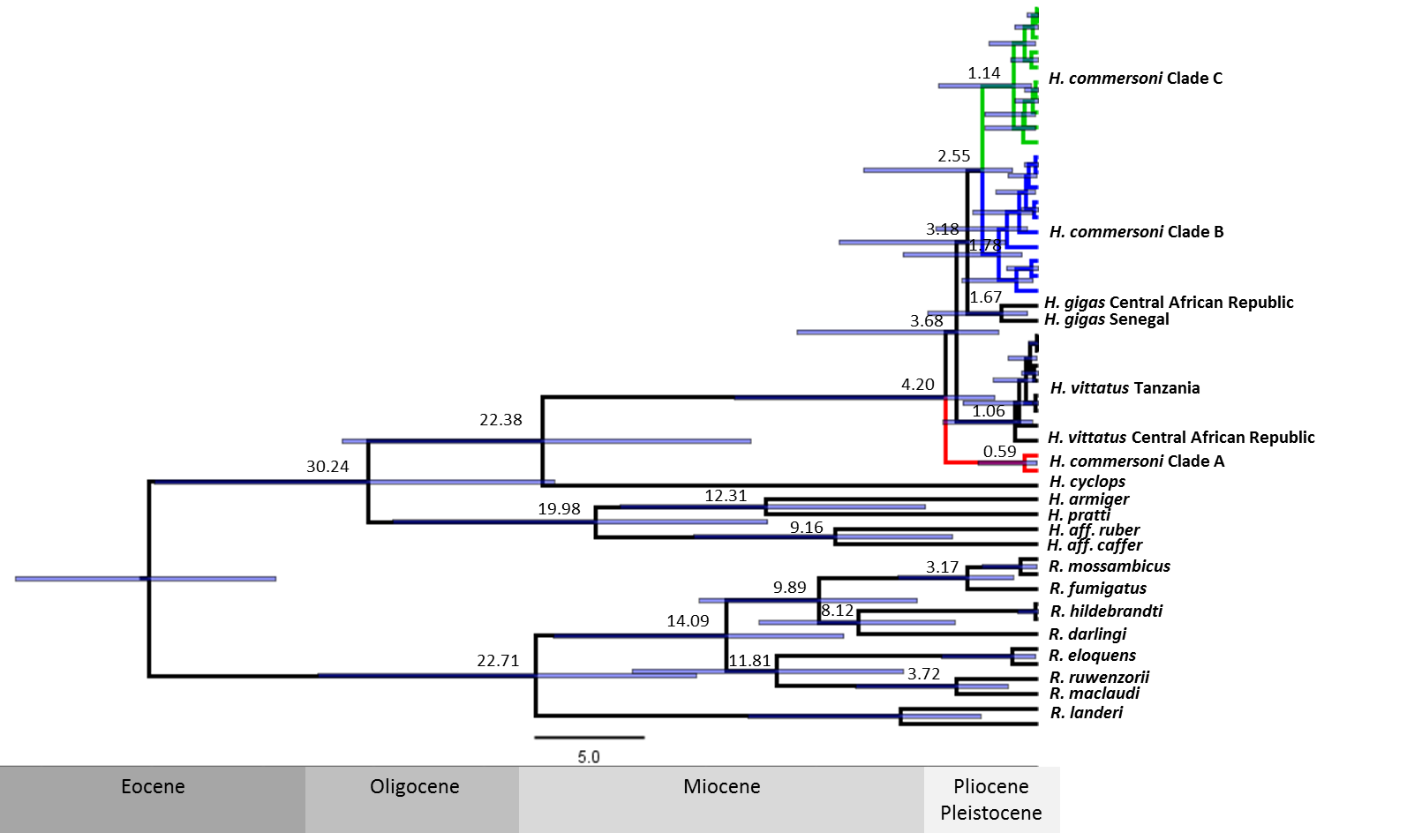

Supplement: Additional file 2: Figure S5. — Alternative maximum clade probability tree, inferred from the analysis of Cyt b data. A strict molecular clock model with a fixed mean substitution rate of 1.30 × 10^-8^ subs/site/year was performed. Values at nodes indicate the posterior mean substitution rate (subs/site/year). Shaded bars indicate the 95% highest posterior density (HPD) credibility intervals. (DOC 118 kb) [file 12862_2015_510_MOESM2_ESM.doc]
